# An automated device for the digitization and 3D modelling of insects, combining extended-depth-of-field and all-side multi-view imaging

**DOI:** 10.3897/zookeys.759.24584

**Published:** 2018-05-17

**Authors:** Bernhard Ströbel, Sebastian Schmelzle, Nico Blüthgen, Michael Heethoff

**Affiliations:** 1 Department of Mathematics and Natural Sciences, University of Applied Sciences Darmstadt, Schöfferstr. 3, 64295 Darmstadt, Germany; 2 Ecological Networks, Technische Universität Darmstadt, Schnittspahnstr. 3, 64287 Darmstadt, Germany

**Keywords:** Focus stacking, morphometry, structure from motion, photogrammetry, 3D modelling, DISC3D

## Abstract

Digitization of natural history collections is a major challenge in archiving biodiversity. In recent years, several approaches have emerged, allowing either automated digitization, extended depth of field (EDOF) or multi-view imaging of insects. Here, we present DISC3D: a new digitization device for pinned insects and other small objects that combines all these aspects. A PC and a microcontroller board control the device. It features a sample holder on a motorized two-axis gimbal, allowing the specimens to be imaged from virtually any view. Ambient, mostly reflection-free illumination is ascertained by two LED-stripes circularly installed in two hemispherical white-coated domes (front-light and back-light). The device is equipped with an industrial camera and a compact macro lens, mounted on a motorized macro rail. EDOF images are calculated from an image stack using a novel calibrated scaling algorithm that meets the requirements of the pinhole camera model (a unique central perspective). The images can be used to generate a calibrated and real color texturized 3Dmodel by ‘structure from motion’ with a visibility consistent mesh generation. Such models are ideal for obtaining morphometric measurement data in 1D, 2D and 3D, thereby opening new opportunities for trait-based research in taxonomy, phylogeny, eco-physiology, and functional ecology.

## Introduction

Digitization has become a major challenge in the curation of natural history collections (Beaman and Cellinese 2012; Berents et al. 2010; Hudson et al. 2015; Mantle et al. 2012; Mathys et al. 2015; Mertens et al. 2017; Moore 2011; Vollmar et al. 2010). Among zoological collections, insects are unparalleled in their number of species and specimens. Hence, automatization of image acquisition and processing seems mandatory for large-scale digitization projects. Whole-drawer imaging (Mantle et al. 2012) allows high-throughput digitization of multiple specimens in one image. The available resolution of approximately 30 µm/pixel, however, seems insufficient for the inspection of delicate morphological details. Furthermore, only the dorsal view is imaged, making lateral and ventral characters inaccessible from the digital data. Hence, this system seems to be well-suited for an online inspection of available specimens in a collection but does not replace physical handling of the specimens for many taxonomical / morphological studies. Images with higher resolution require the specimens to be imaged individually. Due to the small size of many insects, however, images need to be taken with an extended depth of field (EDOF) technique (Brecko et al. 2014). A number of commercially available software solutions allow EDOF image calculation from focus stacks by post-processing (Brecko et al. 2014), and some cameras also have suitable built-in EDOF options (Mertens et al. 2017). In the case of multi-view EDOF imaging, however, time-consuming manual processing is necessary (Nguyen et al. 2014) and available commercial software does not provide well-defined perspectives due to the inability to include information on camera position into EDOF calculation. Hence, large-scale digitization projects use multi-view imaging only when specimens are large enough to keep them in focus as a whole, and use only one or two viewing directions if EDOF images should be needed (Mathys et al. 2015). Sets of images from multiple views do not only provide digital access to more morphological characters, they can also be used to generate colored and textured 3D models of the specimens (Mathys et al. 2013; Mathys et al. 2015; Nguyen et al. 2014; Ströbel et al. 2017). Measurements taken on the virtual 3D models instead of the physical samples protect the delicate specimens and can be easily repeated at any time. Due to the above-mentioned restrictions in combining EDOF and multi-view imaging, however, 3D modelling of higher numbers of small insects seemed impractical (Nguyen et al. 2014).

Traditionally, species descriptions and other scientific or popular documentations focus on 1-D characters, e.g., body length, the length of single body parts or relative length ratios between body parts. However, since organisms have 3D shapes, 2D or 3D traits are meaningful complementary information to characterize body shape and variation more completely (Tatsuta et al. 2017). Hence, phylogenetic analyses should consider morphometrics of shapes and relative landmark positions (Tobias et al. 2010; Wiens 2000). Moreover, many relevant functional morphological traits are in fact 2D or 3D. A ‘functional trait’ represents any kind of phenotypic (morphological, physiological or life-history) characteristic of an organism assumed to influence its performance (McGill et al. 2006). Biodiversity research increasingly focuses on the composition of species’ traits rather than considering only species identities and numbers alone (Loreau et al. 2001; Mouillot et al. 2013; Petchey and Gaston 2002). In the concept of ‘environmental filters’, changes in the environment cause a shift in the distribution of functional traits in species communities (Diaz et al. 1998; Gámez-Virués et al. 2015; Mouillot et al. 2013). Reported changes in morphological trait compositions of insect communities include an increasing relative abundance of species with larger eyes (relative to head width) with increasing land-use intensity (Simons et al. 2016), or decreased forewing length with higher habitat fragmentation (Gámez-Virués et al. 2015). More subtle variation in size and shape across individuals within a species can provide additional insights into environmental responses or resource limitation (Emlen 1997; Peat et al. 2005). Finally, developmental stress in a growing organism may cause asymmetries, e.g., differences between left and right legs, wings, or horns of insects, potentially associated with lower reproductive success or other fitness deficits (Hendrickx et al. 2003; Møller and Thornhill 1998). Hence, many of these functional traits relate to 3D structures, but have been characterized only in 1D or 2D, largely constrained by the availability of methods. In particular, surfaces and volumes are important characteristics that are relevant in a functional eco-physiological context but can only be measured in 3D (Brückner et al. 2017; Kühsel et al. 2017). Therefore, besides archiving and digitizing museum collections, trait-based research areas in functional ecology, eco-physiology, and evolutionary biology would greatly benefit from easily available 3D scanning techniques.

Established tomographic 3D techniques have some shortcomings when it comes to pinned insects. Although delivering landmark data with a high precision (e.g., Betz et al. 2007; Heethoff and Norton 2009; Schmelzle et al. 2009), X-ray micro-tomography requires costly equipment or access to very limited beamtime at suitable synchrotron facilities. Furthermore, high X-ray attenuation of the needle compared to the insect body results in distracting artifacts. Finally, as pinned insects are dried without preserving their inner organization, tomography seems of limited value, also since it does not recover color/texture of the specimen. Hence, the use of visible light in combination with real color imaging seems the most adequate technique for digitizing insect collections.

Visible light is to some extent reflected or remitted at or near the surface of the insect. Light returning from the specimen bears information about position, reflectivity, and color, and forms the basis for optical 3D surface scanning. Triangulation techniques determine the spatial position of surface points by the intersection of light rays. In the case of passive triangulation, ambient illumination is applied, and all rays used for measuring are viewing rays of different cameras or of a camera in different positions. Two such techniques, ‘Structure from Motion’ (SfM) and ‘Shape from Silhouette’ (SfS), are suitable for 3D insect scanning (Mathys et al. 2013; Nguyen et al. 2014).

SfS (Cheung et al. 2005; Furukawa and Ponce 2009; Gallo et al. 2014) is based on viewing rays tangential to the object (see Suppl. material 1: Fig. S1). These enclose visual cones with respect to the projection centers of the cameras. The object surface is approximated by the ‘visual hull’, the common intersection of all visual cones. Resulting 3D models need to be calibrated by scaled markers included in the images. Moreover, since there are no silhouette rays from concave parts of an object, it is not possible to reconstruct indentations on insect bodies. Until now, the only published device for capturing natural-color 3D models of pinned insects is based on SfS (Nguyen et al. 2014). Estimated as a ‘proof of concept’ by the authors, this pioneering system used a DSLR camera and a two-axis rotating table. The camera calibration was carried out with the aid of a mat with printed markers, imaged together with the insect pinned on this marker mat. In consequence, only a part of the sensor area was available for the specimen. Moreover, the arrangement of specimen and mat precludes imaging the underside of the insects. The authors propose to either mount the specimens with an auxiliary second pin or to re-pin the insects. This is a major shortcoming of the SfS setup since re-pinning bears an ultimate risk of damaging the specimen, and probably no museum would agree to re-pin any type specimens from their collection.

SfM (Seitz et al. 2006; Szeliski 2011; Ullmann 1979; Westoby et al. 2012) is a photogrammetric technique that uses images from different viewing directions (i.e., different camera positions with respect to the specimen or different specimen poses with respect to the camera) and identifies corresponding feature points on these images (see Suppl. material 1: Fig. S1). Unlike traditional photogrammetry, SfM does not need a previous calibration of camera positions and orientations, since these are determined together with the object structure (simultaneous calibration). This becomes possible by the high number of corresponding feature points that can be detected in overlapping images of well-textured objects such as insect specimens - an approach that has been boosted by the emergence of efficient new algorithms for feature detection and matching (e.g., SIFT, Lowe 2004), powerful graphics processor units, and user-friendly software (Agisoft PhotoScan), including generation of 3D-models with visibility-consistent meshing (VCM) techniques (see also: Aroudj et al. 2017; Vu et al. 2012).

Here, a new imaging device (the Darmstadt Insect SCanner: DISC3D) is presented that overcomes the restrictions of the above-mentioned EDOF multi-view imaging, and provides data suitable for both, digitally archiving insects (and other small objects) and generating 3D models. We developed DISC3D with the aim of affordability, clonability, and minimization of manual processing steps. DISC3D allows specific configurations for different requirements (e.g., high resolution for archiving, high number of views for 3D modelling of complex structures, or fast imaging for mass digitalization).

## Materials and methods

**Figure 1.**
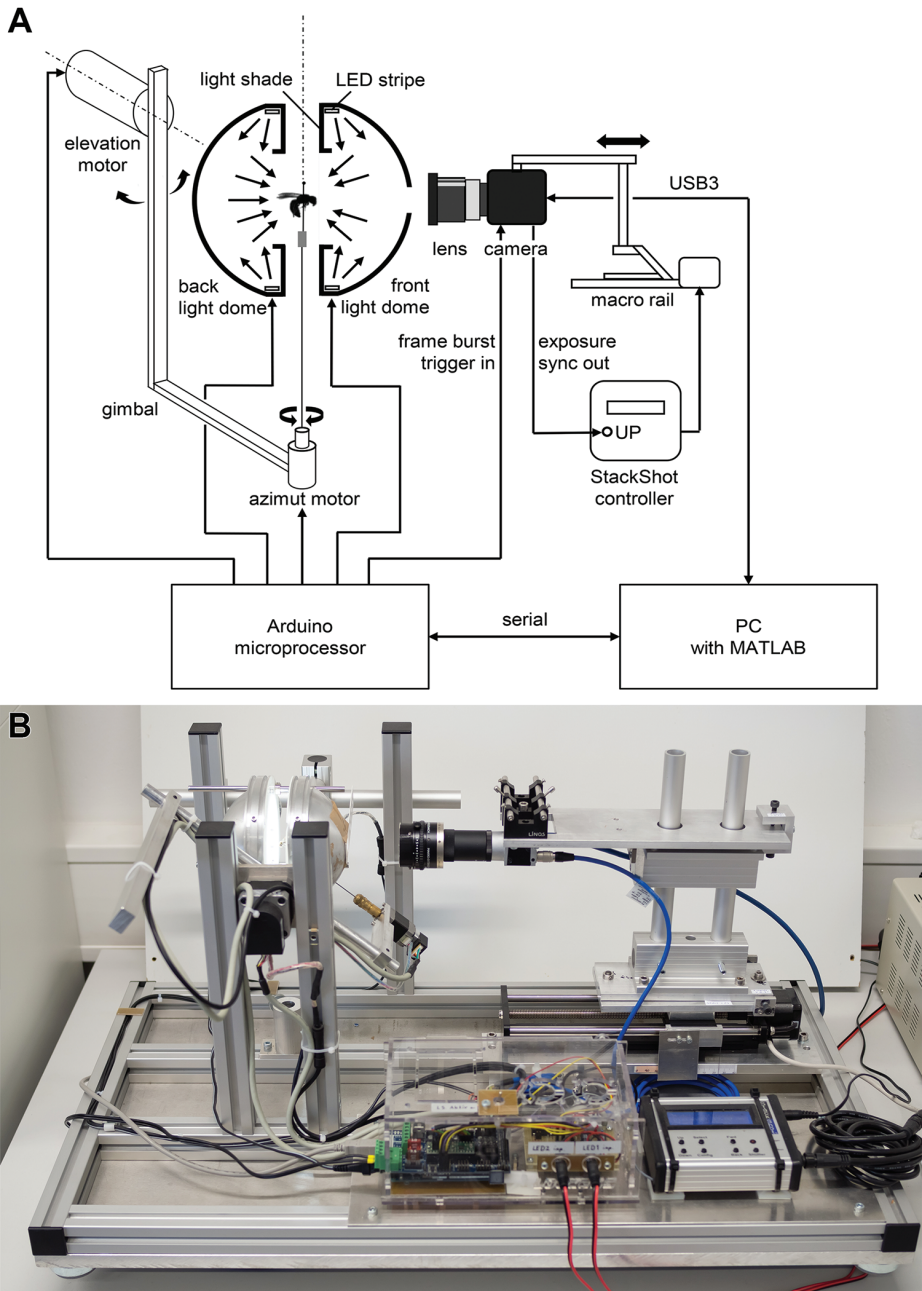
Schematic setup (**A**) and image (**B**) of the Darmstadt Insect Scanner DISC3D.

DISC3D is published under the Creative Commons license CC BY-SA (https://creativecommons.org). The total costs of the device range between 4,000€ and 8,000€ (depending on the camera and availability of computers and educational software licenses). In the following an overview is given of the components of DISC3D (Fig. 1) and the measuring process. Detailed technical information and calculations can be found in the supplement. Visit also www.econetlab.net/disc3d for current developments.

### Animals used in the study

For demonstration of DISC3D, a set of pinned insects and snail shells from our collection with a representative shape range and sizes between 1.5–30 mm were chosen. The following species were included:

Coleoptera:

*Prosopocoilus savagei* Hope (23 mm)

*Anoplotrupes stercorosus* Scriba (21 mm)

*Stenocorus meridianus* L. (19 mm)

*Typhaeus typhoeus* L. (18 mm)

*Rutpela maculata* Poda (17 mm)

*Valgus hemipterus* L. (8 mm)

*Cryptocephalus sericeus* L. (7 mm)

*Pogonocherus hispidus* L. (6 mm)

*Phyllobius pyri* L. (6 mm)

*Tytthaspis sedecimpunctata* L. (3 mm)

Lepidoptera:

*Zygaena filipendulae* L. (15 mm body size, 30 mm wingspan)

Hymenoptera:

*Paraponera clavata* Fabricus (20 mm)

*Osmia adunca* Panzer (12 mm)

*Sphecodes ephippius* L. (8 mm)

Diptera:

*Thricops* sp. Róndani (8 mm)

*Culex pipiens* L. (5 mm)

*Oscinella frit* L. (1.5 mm)

Gastropoda:

*Helicodonta obvoluta* O.F. Müller (9 mm)

*Aegopinella nitens* Michaud (8 mm)

*Discus rotundatus* O.F. Müller (5 mm)

### Specimen mounting and orientation

A black foam plastic adapter is used to connect the insect pin to a ca. 100 mm long and 1 mm thick supporting steel pin. The mounting is adjusted to the center of rotation of a two-axis motorized gimbal and allows a free view on the specimen from virtually all sides. During an insect scan, the specimen undergoes a preassigned ‘pose program’ with an approximately constant angular distance between neighboring poses. Pose programs can be adjusted for specific needs (e.g., shape and complexity of the object). In this study, we mostly used a standard pose program with a mean angular distance of 10° of two neighboring poses, and a total number of 412 poses, 14 of which are not accessible due to geometric and optical constraints of the sample holder (see Suppl. material 1: Fig. S2). For repeatability of the poses, the stepper motors of the gimbal are reinitialized before every insect scan.

### Illumination

**Figure 2.**
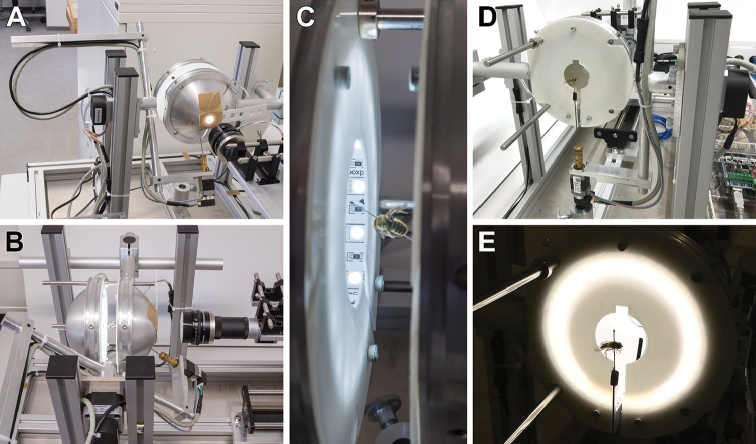
Illumination by two hemispherical white-coated domes (**A–C**). The back-light-dome can be removed for specimen mounting (**D, E**). No direct light from the LED-stripes hits the specimens (**C, E**).

Many insect surfaces are glossy and show bright reflections (‘hotspots’) when illuminated directly by a point-shaped light source. For that reason, we use two separately addressable hemispherical illumination domes: a ‘front-light dome’ that is adjacent to the camera but averted, and a ‘back-light dome’ on the far side facing the camera (Fig. 2). Each dome is equipped with a dimmable white LED strip attached to its inner circumference and a light shade extending into the dome that prevents direct illumination of the samples. Through backscattering by the white coated inner surface of the dome, illumination of the samples is indirect and nearly homogeneous. Both the front-light and the back-light dome feature a slot to accommodate the supporting pin when tilted by the gimbal. The front-light dome additionally features an opening with an exchangeable aperture for the camera, whereas the back-light dome can be completely removed to easily switch samples between scans.

The indirect front-light illumination of the sample is optimal for curved or bumpy glossy insect surfaces. The back-light dome produces an even background illumination, and with the front-light switched off, a silhouette image of the insect can be captured. This silhouette image can be used to mask the background in the images taken with front-light illumination.

### Optics and focus stacking

**Figure 3.**
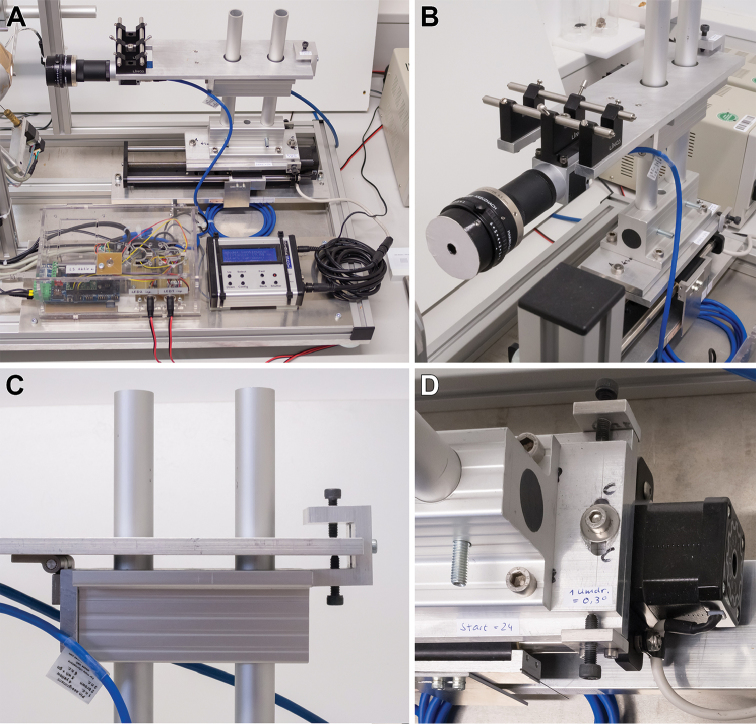
The camera is mounted on a macro-rail (**A**). The camera position and orientation can be fine-tuned in all directions (**B–D**). The camera lens is covered by a pinhole-cap (**B**).

DISC3D is equipped with an industrial camera and a compact macro lens (Fig. 3). The main advantages of this configuration over a DSLR camera system are the excellent angular accessibility to the specimen, the absence of a mechanic shutter, and full computer control of both image acquisition and processing. The detailed criteria for the choice of the optics and further technical data can be found in the Suppl. material 1. To extend the shallow depth-of-field of the insect images, a video stream of images is captured under front-light condition as the camera moves forward (towards the specimen), with the focal plane crossing the whole insect body. The result is a stack of images which are later merged for a front-light EDOF-image, a technique known as ‘focus stacking’ or ‘z-stacking’ (see Suppl. material 2). As the camera moves back to the starting point, another video stream is taken under back-light condition,. The resulting back-light EDOF image serves for the segmentation of the background. The motion of the camera along its axis is accomplished by a motorized macro rail system (Cognisys StackShot^TM^, Cognisys Inc., Traverse City, USA; see Suppl. material 1: S3).

### Image acquisition and processing

The complete process of image acquisition and processing is controlled with MATLAB (Mathworks, Natick, USA). Camera configuration and start, as well as the readout of the images are accomplished via the USB3 Vision interface standard. An Arduino Mega 2560 microcontroller board, connected to the PC via a serial interface controls the motion of the gimbal motors, switches the LEDs, and triggers the video stream of the camera. To synchronize the macro rail controller with the video stream, the StackShot motion is triggered by the ‘exposure active’ camera output signal of the first video frame.

**Figure 4.**
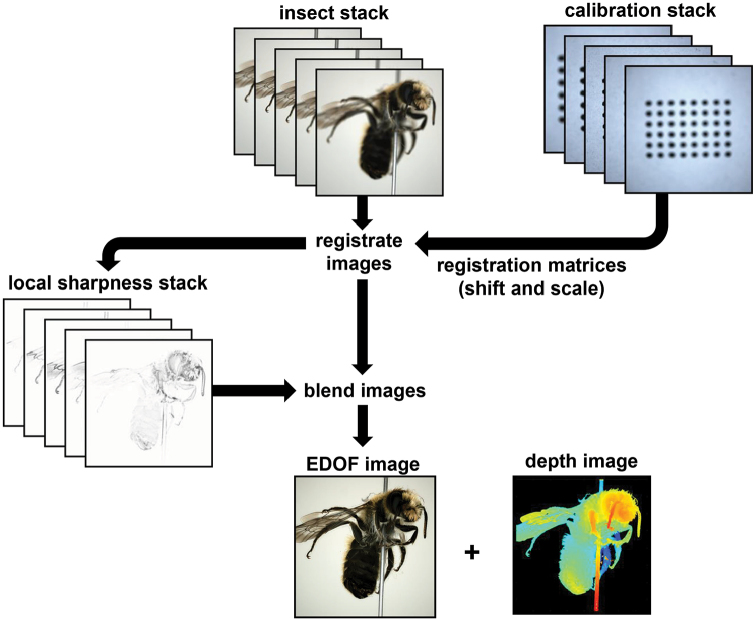
Workflow of EDOF-calculation. For a detailed description, see Suppl. material 1: S2.

For the calculation of the EDOF image from the focus stack, several proven software products are available (Brecko et al. 2014). Nevertheless, we decided to develop our own MATLAB code for the following reasons: (i) the incurring raw images can be processed in parallel to the scan, avoiding the transfer of a large amount of data to a different software; (ii) the high repeatability of the macro rail motion allows calibrating the focus stack acquisition, an option which considerably accelerates the later EDOF image calculations but is not supported by standard focus stacking software; (iii) to ensure that EDOF images can be used for photogrammetry, they must comply with the pinhole camera model, i.e., all parts of the images must be consistent with a unique perspective (Luhmann et al. 2013). The EDOF images produced by standard focus stacking software usually do not meet this condition. The process of EDOF image calculation on the base of the calibration of the focus stack acquisition is illustrated in Fig. 4 and fully described in the Suppl. material 1: S4, see also Suppl. material 2: SV1.

**Figure 5.**
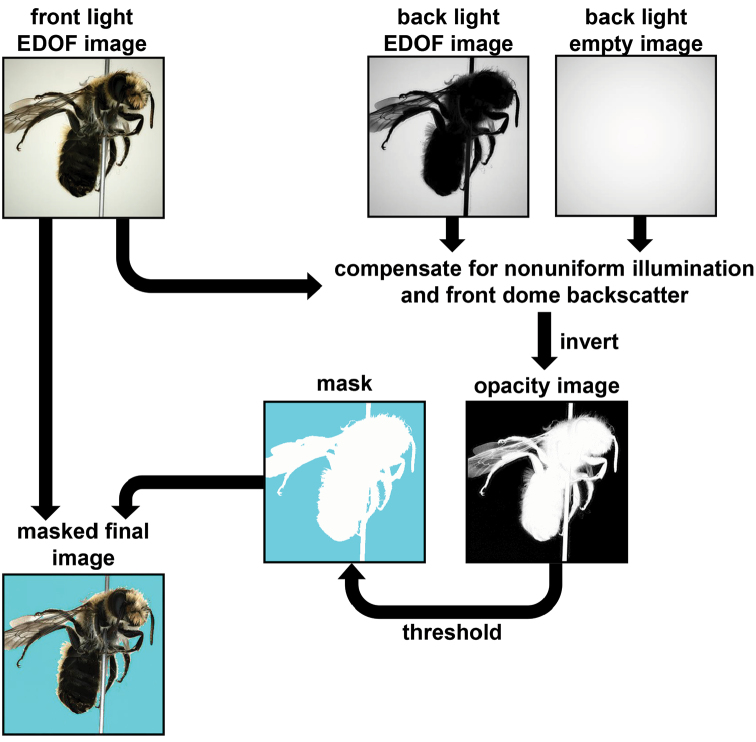
Workflow of image masking using front- and back-light information.

After focus stack processing of the front-light and back-light images, a binary mask for the segmentation of the background is calculated and saved in the alpha-channel of the front-light image (Fig. 5; see also Suppl. material 1: S5). Up to half a terabyte of raw image data are acquired during an insect scan with 10° mean angular distance between the poses. Processing results in storage of 398 masked images in the lossless PNG file format with a total of 300–600 MB. Depending on the size of the insects, the total scanning time including stack processing ranges from two to five hours. Because only about 40–100 min are needed for the actual measurement (image acquisition), faster image processing could considerably accelerate the scans. Other options to reduce the scan time are discussed in the Suppl. material 1: S6.

The MATLAB code is menu-based and includes (i) several calibration functions, (ii) the control of insect positioning, front- and backlight illumination, camera exposure and gain parameters, (iii) the interactive adjustment of several EDOF calculation and masking parameters on the base of the quality of obtained images, and (iv) the start of the scan with the chosen pose program and evaluation range of the image stacks. The interactive adjustment allows customizing all parameters to the specific characteristics of the particular specimen. Alternatively, proven parameter sets from earlier scans of the same or similar specimens can be used. The scan itself is completely automatic.

### 3D modelling

**Figure 6.**
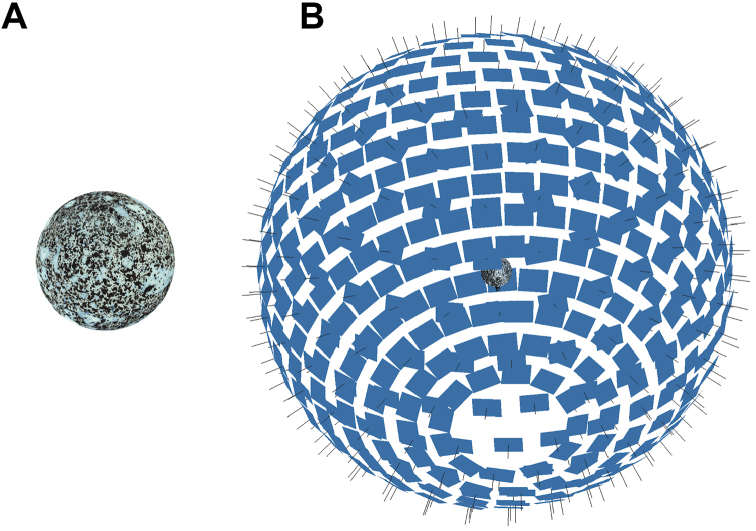
Calibration sphere (**A**) and camera positions estimated in PhotoScan Pro (**B**).

After the scan, the masked EDOF images of all poses are transferred to the SfM software PhotoScan Pro 1.4 (Agisoft LLC, St. Petersburg, Russia). Depending on shape and surface texture of the insects, images of some poses may not contain a sufficient number of matching feature points to allow the simultaneous calibration of the respective camera positions. Such weak poses are discarded by PhotoScan Pro, which impairs the quality of the resulting model. To cope with this problem, and to accelerate the SfM calculation, a special 3D target with a high number of features (textured sphere, see Fig. 6) is used for a “pose calibration”. Thanks to the high repeatability of the motorized gimbal, the camera data (internal parameters, positions, and orientations) found with this sphere target can be used as an approximation in later insect scans with the same pose program. Additionally, the known diameter of the sphere can be compared to the resulting 3D model to verify the correct scaling of the model (see Suppl. material 1: S7). PhotoScan Pro allows further optimization of these camera data, thus considerably improving the quality of the resulting 3D point cloud. Our standard workflow with PhotoScan Pro is: (i) add images; (ii) import masks; (iii) import camera positions from the pose calibration and calculate point cloud (sparse cloud); (iv) optimize camera positions; (v) build (and refine) mesh; (vi) build texture; (vii) export 3D-model in desired format. Further automatization could be achieved by a python script including most if not all steps.

For animation (see Suppl. material 3: SV2), 3D-models (meshes) and textures of scanned specimens were exported in the Wavefront OBJ format, imported into Blender (www.blender.org) and rendered with a lighting model suitable to mimic a semi-natural appearance of surface structure and reflections. Resulting animated videos were edited with the free software Shotcut (Meltytech, LLC.). PhotoScan Pro can also export 3D-models directly into 3D-PDFs and several other 3D formats.

### Morphometry

Some of our 3D models were used to demonstrate the ease of obtaining 3D morphometric data of the scanned specimens in PhotoScan Pro. Surface area and volume were measured and plotted against each other.

Additionally, the reliability of measurements taken on 3D models was tested by comparing morphometric data obtained from the model and on the specimen itself using the statistical analysis software PAST (Hammer et al. 2001). The width of the scutellum (*sc*) and the length of the tibia (*ti*) of the left hind leg were measured on a specimen of the dung beetle *Anoplotrupes stercorosus*, using both, a Keyence VHX-5000 digital microscope equipped with a Z20 lens, and on a 3D-model of the same specimen taken with DISC3D, using the measuring tools in PhotoScan Pro.

## Results and discussion

The quality of the images and the 3D models of DISC3D is demonstrated here by some illustrative examples of insects and snail shells. We present some models as animated videos and some as textured or non-textured polygon-models, implemented as interactive 3D content.

### Archiving multi-angle EDOF images

**Figure 7.**
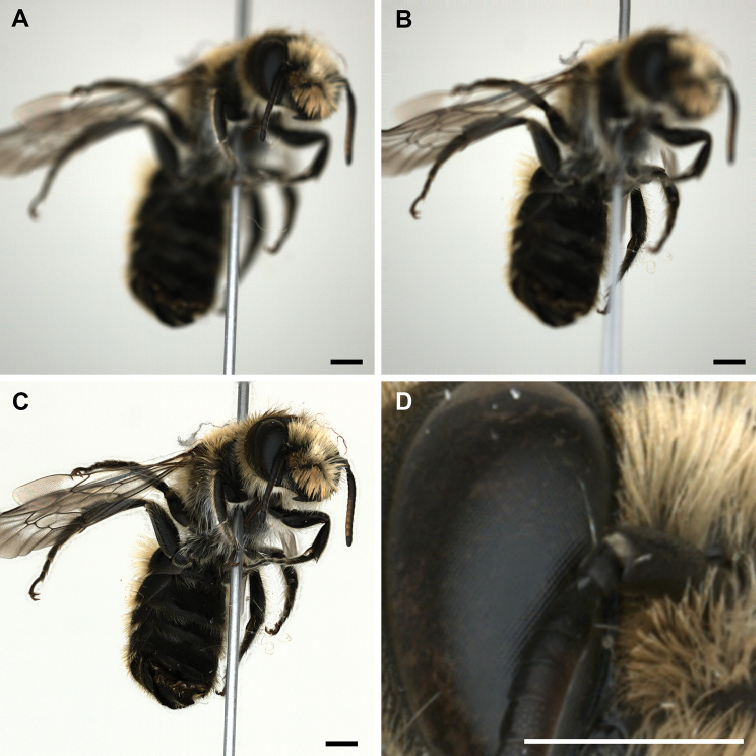
*Osmia adunca*, two exemplary raw images of the front-light stack, with the focal plane going through the proximal (**A**) and the distal part (**B**) of the sample, the EDOF image (**C**), and a detail of the latter (**D**) demonstrate the resolution. Scale bars: 1 mm.

**Figure 8.**
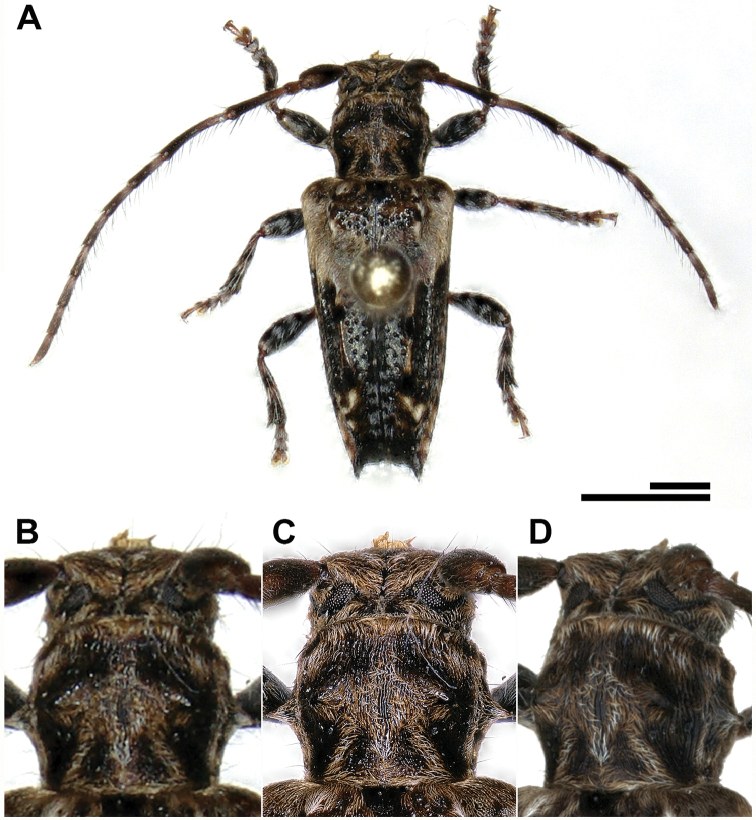
Comparison of images taken with a Keyence VHX 5000 digital microscope (lens: Z20, **A–C**) and DISC3D (**D**). The whole specimen of *Pogonocherus hispidus* can be imaged at once with the VHX 5000 with a X30-magnification (**A**). To compare the digital resolution, we focus on the pronotum of the beetle (**B**: VHX 5000, ×30; **C**: VHX 5000, ×100, **D**: DISC3D, ×1.26). Scale bars: 1 mm.

DISC3D allows automatic acquisition of multi-view EDOF images for digitization and digital archiving of pinned insects. There is a trade-off between the digital resolution (size of the pixels on the side of the insects), and the number of views on the one hand, and the scanning time on the other hand. The quality and digital resolution of the EDOF images created by our stacking algorithm is well suitable for the inspection of many relevant morphological features and avoids artifacts of other approaches (Nguyen et al. 2017). Industrial cameras with higher resolution could also be used (e.g., Basler acA4600-10uc with 14 MP), but would lead to larger file sizes, longer image processing times (see Suppl. material 1: S2), and longer times for 3D reconstruction. Hence, we think that the 4 MP camera we used is a good compromise of image quality, resolution, and processing time for many needs in insect imaging (see Figs 7, 8). The absence of spot-like specular reflections (‘highlights’, cf. Fig. 8C, D) is unusual in macro photography and gives our EDOF images a somehow ‘flat’ appearance. However, this effect results from the ambient dome illumination and prevents lighting artifacts from being misinterpreted as insect features.

### SfM workflow

**Figure 9.**
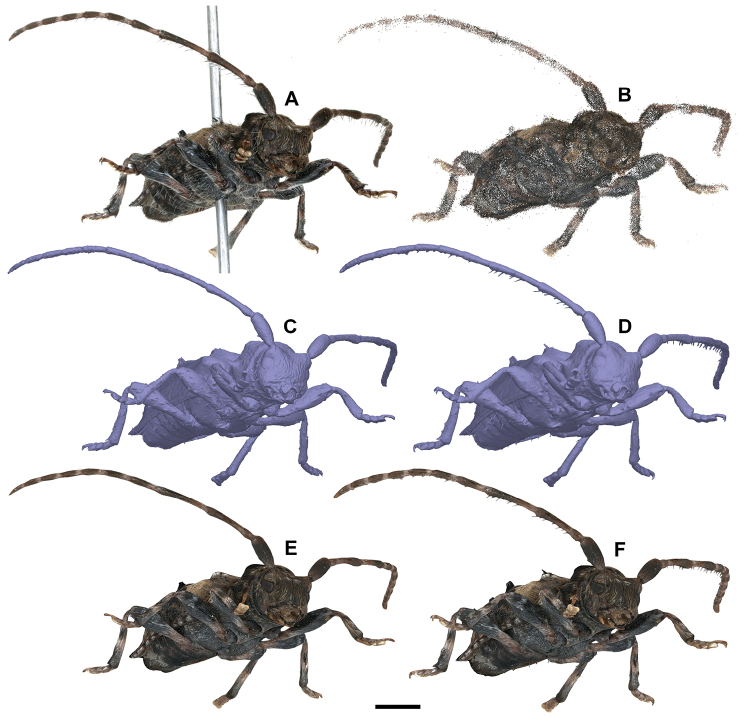
Workflow of model generation of *Pogonocherus hispidus* with PhotoScan Pro. In total, 398 EDOF-images are taken with DISC3D (one example is shown in **A**). Using the image data, masks and camera positions estimated with the calibration sphere (see Fig. 6), a sparse cloud with optimized camera positions is generated (**B**). Two options for model generation are available: direct mesh calculation based on a dense point cloud (**C**) or meshing with visual consistency (**D**). Resulting meshes can be textured (**E, F**). Scale bar: 1mm.

**Figure 10.**
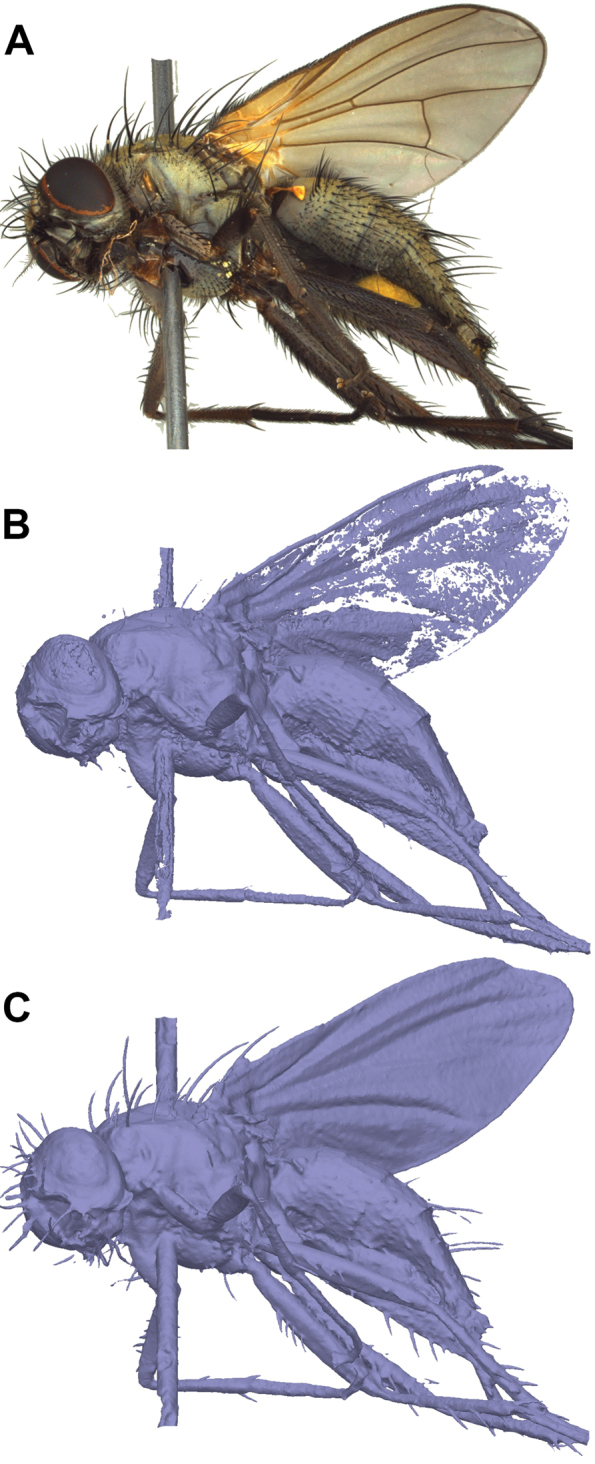
Comparison of dense-cloud-based mesh generation and visual consistency meshing of *Thricops* sp. (**A** EDOF image). Thin and delicate structure like wings and setae are not well modelled from the dense cloud (**B**) but well preserved by visual consistency meshing (**C**).

**Figure 11.**
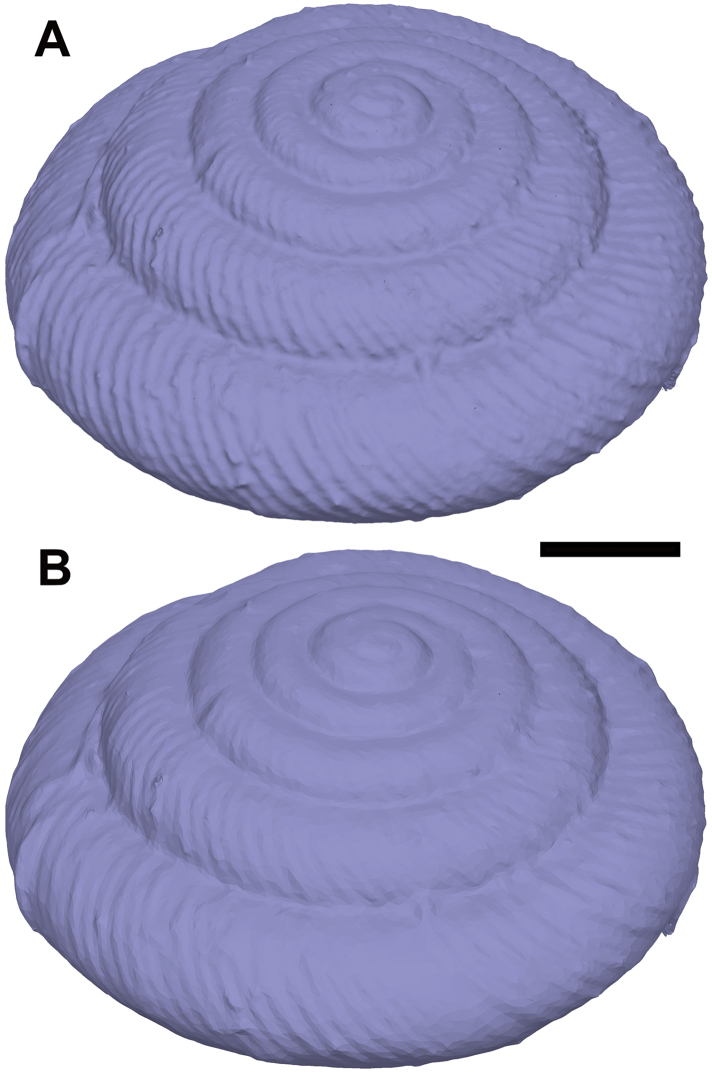
Comparison of mesh quality and number of polygons, exemplified by a model of the shell of *Discus rotundatus*. The model with 1 million faces (**A**) has a file size (3D-PDF) of 35 MB and shows more detail, but the reduced model with 75.000 faces (**B**) still well resembles the structure with a file size (3D-PDF) of only 3 MB.

All 3D models shown here have been generated with Agisoft PhotoScan Pro with visibility-consistent mesh generation enabled. We show the results of the main steps, exemplified by a scan of a 6 mm long specimen of the Lesser Thorn-tipped Longhorn Beetle *Pogonocherus hispidus* (Fig. 8). Image data were taken with a magnification of 1.26 (resulting in a digital resolution of 4.37 µm). After importing the 398 EDOF images, the masks from the alpha channel, and the external camera parameters from the pose calibration with the textured sphere, the sparse cloud was generated with highest accuracy settings, and finally the camera externals were optimized with respect to the actual data (Fig. 9B). The optimized alignment can either be used for the generation of a dense cloud to be meshed into a closed surface model (Fig. 9C) or for VCM generation (Fig. 9D). VCM is a new (and experimental) feature of PhotoScan Pro 1.4. However, since the time needed to generate a meshed model was considerably shorter (0.5–2 h for VCM vs 4–8 h for dense cloud calculation), and delicate structures (e.g., setae, wings) turned out to be modelled much better (Figs 9, 10), we exclusively used this option. Finally, meshes were smoothed, reduced to 75.000 faces (polygons) and textured, using the ‘mosaic’ option and a 2500*2500 pixel texture atlas. Most models initially had several hundred thousand (up to a few million) faces, with a higher spatial resolution of the models, but also larger files. The reduction to 75.000 faces causes an acceptable loss of detail in most cases while keeping the file size of the model small. The difference is illustrated for the shell structure of the snail *Discus rotundatus* O.F. Müller (5 mm shell diameter, Fig. 11).

### Illustrative examples

**Figure 12.**
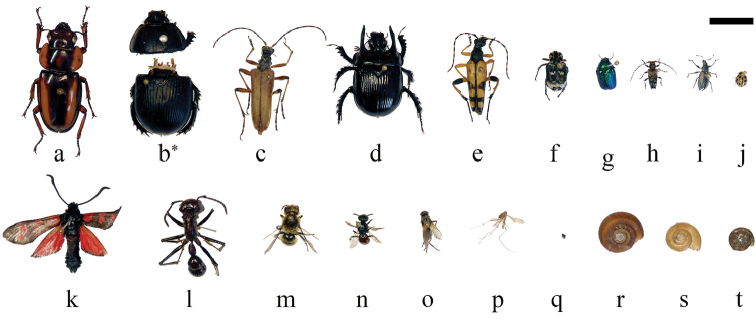
Overview and size comparison of the specimens used in this study. Coleoptera: **a** *Prosopocoilus savagei* **b** *Anoplotrupes stercorosus*, *: specimen was broken during comparative measurements **c** *Stenocorus meridianus* **d** *Typhaeus typhoeus* **e** *Rutpela maculata* **f** *Valgus hemipterus* **g** *Cryptocephalus sericeus* **h** *Pogonocherus hispidus* **i** *Phyllobius pyri* **j** *Tytthaspis sedecimpunctata*; Lepidoptera: **k** *Zygaena filipendulae*; Hymenoptera: **l** *Paraponera clavata* **m** *Osmia adunca* **n** *Sphecodes ephippius*; Diptera: **o** *Thricops* sp., **p** *Culex pipiens* **q** *Oscinella frit*; Gastropoda: **r** *Helicodonta obvoluta* **s** *Aegopinella nitens* **t** *Discus rotundatus*.; Scale bar: 1 cm (keep in mind that not all specimens are equidistant to the lens; i.e., at the same height of the needle).

A set of insect and snail specimens were chosen with a representative shape and size range of 1.5–30 mm for visualization (Fig. 12 and Suppl. material 3: SV2).

**Figure 13.**
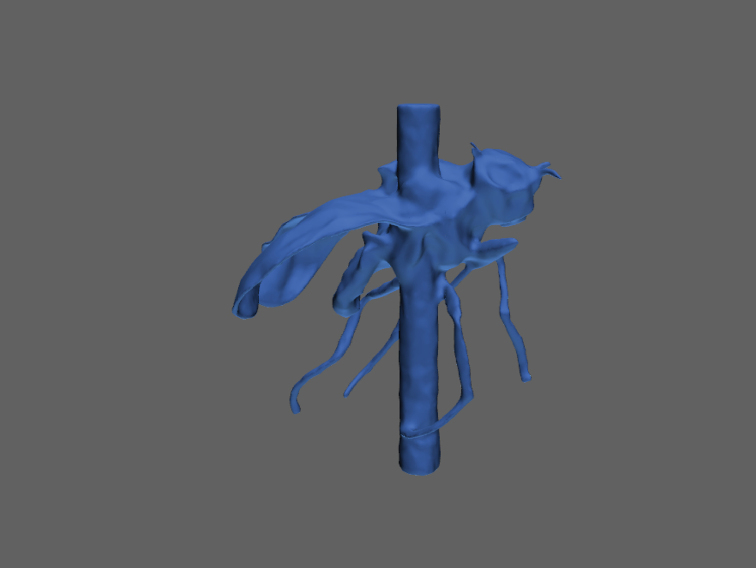
Interactive 3D-model of *Oscinella frit* (1.5 mm body size).

With 1.5 mm, *Oscinella frit* is probably the smallest object that has ever been 3D modeled by SfM techniques (Fig. 13). A thin insect pin of size 000 with a diameter of 0.25 mm has been used for preparation, and it becomes obvious that smaller insects could hardly be pinned, even with the thinnest pins with a diameter of 0.1 mm, without strongly distorting the shape of the specimen. This could to some extend be circumvented by carefully gluing samples onto the needles, as demonstrated here for the snail shells. Since our sample holder is designed for pinned samples, the device seems well suitable for even the smallest specimens that can be prepared by pinning.

**Figure 14.**
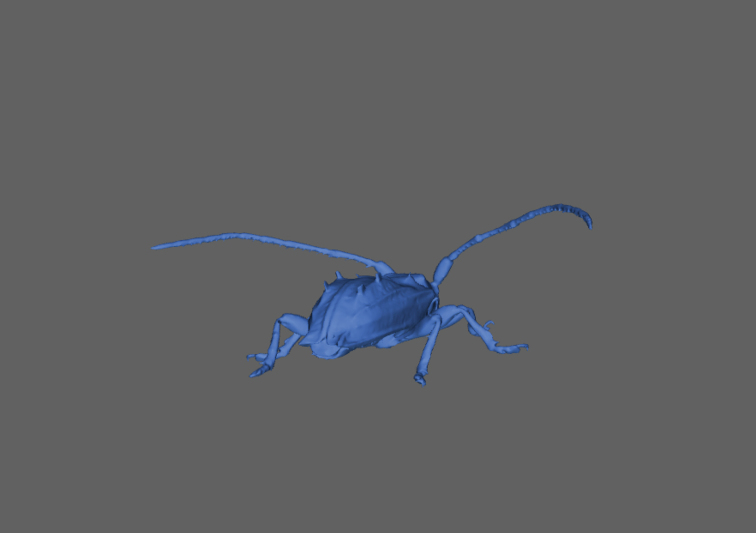
Interactive 3D-model of *Pogonocherus hispidus* (6 mm body size).

**Figure 15.**
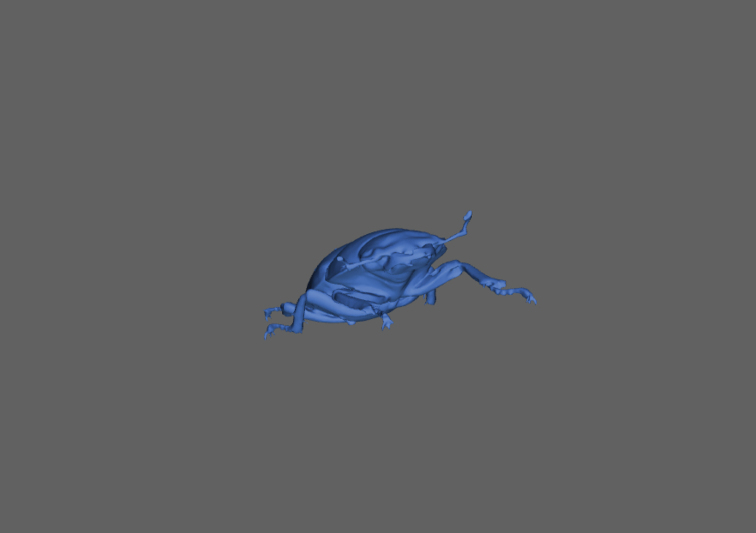
Interactive 3D-model of *Prosopocoilus savagei* (23 mm body size).

We further provide interactive 3D-models of a mid-sized (*Pogonocherus hispidus*, 6 mm, Fig. 14) and a large beetle specimen (*Prosopocoilus savagei*, 23 mm, Fig. 15). The interactive 3D figures allow measuring certain morphometric parameters with the measuring toolbox.

**Figure 16.**
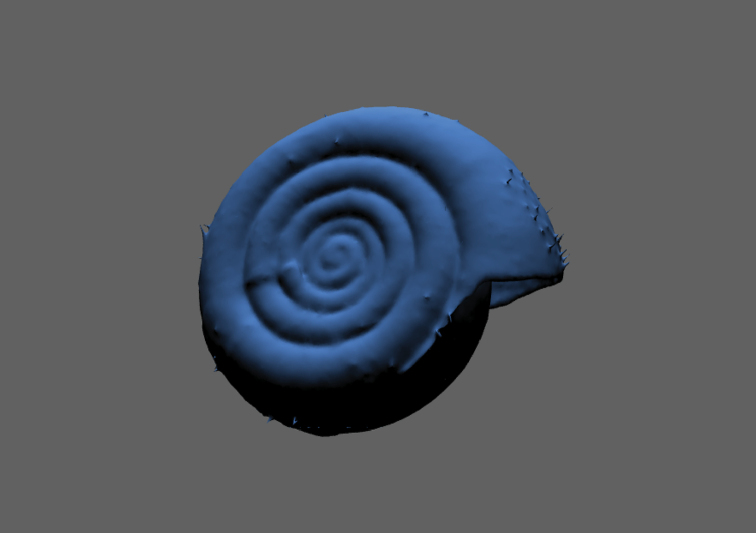
Interactive 3D-model of *Helicodonta obvoluta* (9 mm shell diameter).

The models generated with EDOF images obtained by DISC3D have several advantages when compared to the models published by (Nguyen et al. 2014): (i) specimens can be scanned without being re-pinned; (ii) the synchronous process of image acquisition and EDOF calculation allows full automatization; (iii) SfM enables modelling of even deep indentations, which could be demonstrated with the umbilici and apertures of the snail shells (Fig. 16; see Suppl. material 3: SV2).

### Morphometry using 3D models

**Figure 17.**
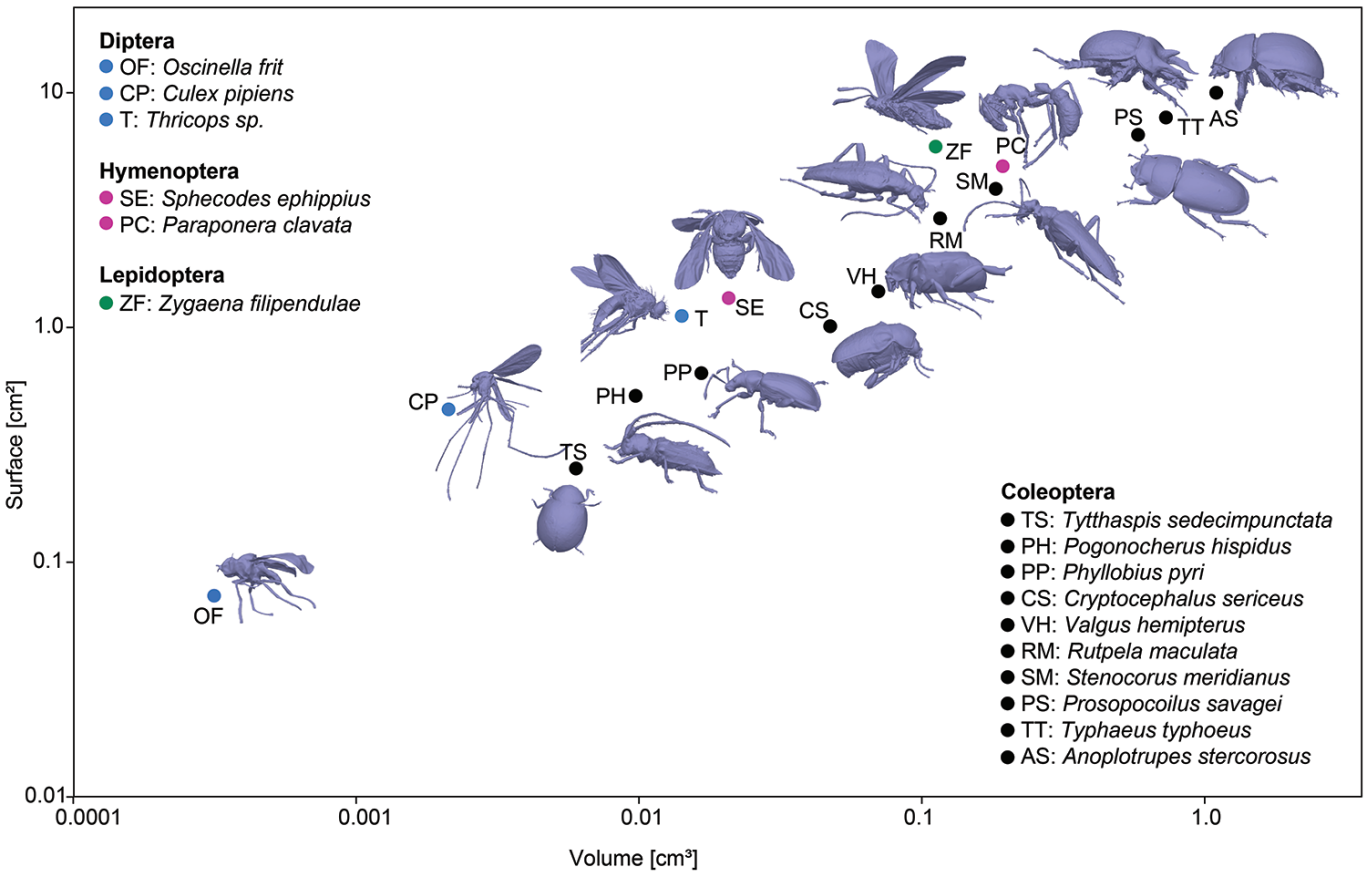
Relation of surface area and volume for all insect species presented here. While the dipteran, hymenopteran and lepidopteran species had their wings unfolded, all beetles had the wings folded underneath their elytra. Models are not to scale.

Surface area and volume of the 3D models were measured in PhotoScan Pro and plotted against each other (Fig. 17). The surface areas and volumes range from 0.072 cm² and 0.00031 cm³ (*Oscinella frit*) to 9.99 cm² and 1.0986 cm³ (*Anoplotrupes stercorosus*), respectively. Some specimens had unfolded wings, generally leading to higher surface areas. The biological consequences of SA/V ratios will not be discussed here (we just aimed at demonstrating the ease of measuring), but they affect physiological processes and can influence performance and distribution of species (Kühsel et al. 2017).

**Figure 18.**
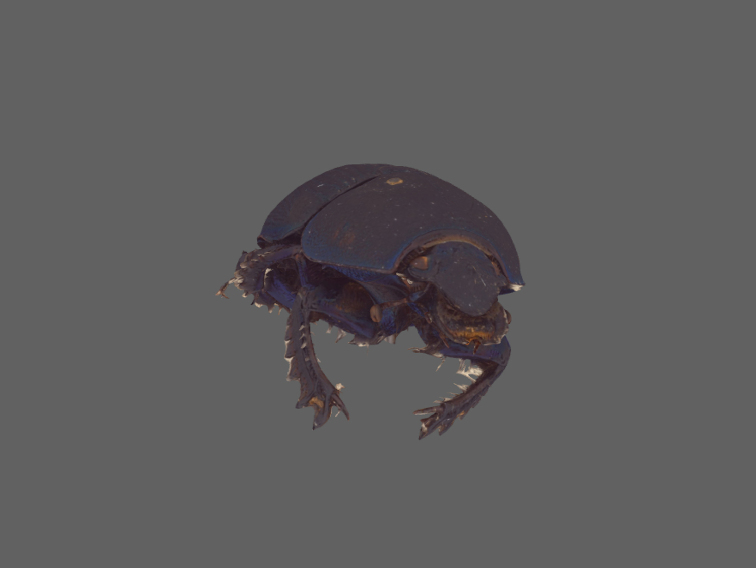
Interactive, textured 3D-model of *Anoplotrupes stercorosus* (21 mm body size).

3D models are not only helpful to obtain data which are impossible to be measured on the specimen itself (like surface area), they may also help to obtain more reliable data even of 1D measurements by avoiding parallax errors. We asked 22 people (laypersons, students and skilled entomologists) to measure two simple distances between easily observable landmarks that differed in their susceptibility to parallax errors (width of scutellum (*sc*) and length of the tibia (*ti*) of the left hind leg) on a specimen of the dung beetle *Anoplotrupes stercorosus*, using both, a Keyence VHX-5000 digital microscope (=2D), and a 3D-model (=3D) of the same specimen (Fig. 18). Even with the ‘easy’ measurement of *sc*, we found remarkable differences in the measurements (in mm: 2D: mean 2.63, SD: 0.21, range: 2.32–3.11; 3D: mean 2.72, SD: 0.07, range: 2.58–2.87) which were even greater regarding *ti* (in mm: 2D: mean 6.11, SD: 0.51, range: 5.24–6.88; 3D: mean 6.61, SD: 0.07, range: 6.45–6.73). There was a strong person-effect for measurements taken with the microscope (Friedmann-test; 2D: χ² = 75, p < 0.001, df = 21), but not for measuring on the 3D model (Friedmann-test; 3D: χ² = 27, p = 0.14, df = 21), indicating that less training and care are necessary when measuring on the 3D model. Moreover, morphometric data measured on the 3D model were more reliable: the coefficient of variance (CV) of individual 2D microscopic measurements was more than three times higher for *sc*, and more than seven times higher for *ti* , as compared to 3D model measurements (*sc*: CV(2D) = 8.0%, CV(3D) = 2.5%, F-test: F = 9.87, p < 0.0001; *ti*: CV(2D) = 8.3%, CV(3D) = 1.1%, F = 49.69, p < 0.0001).

Due to observer errors, morphometric data can vary by more than 14% of the mean measured value (Viscardi et al. 2010), leading to the introduction of noise and potential bias when compiling composite datasets. We also observed a maximum deviation of 14% from the mean value of both, *sc* and *ti* when using the microscope. This error was strongly reduced to 5.5% (*sc*) and 2.4% (*ti*) when using the 3D model. Hence, morphometric measurements taken on 3D models do not only allow access to ‘new’ data (e.g., surface areas), they also provide more reliable and less error-prone data.

## Conclusions

To the best of our knowledge, DISC3D is the first and only system for automated multi-view EDOF imaging. The system allows the digitization of natural history collections and effective exchange of information about a specimen, avoiding physical handling or transfer of the specimen itself. The 3D-models facilitate accurate reproducible 1D, 2D, and 3D measurements to characterize the specimen, including functionally relevant traits such as surfaces or volumes, a promising perspective for functional ecology, comparative zoology, and physiology. For large scale digitization projects, several scanners could be used simultaneously. Due to the high degree of automatization, one person should be able to operate up to five devices in parallel for scanning and 3D-modelling. We encourage the community to copy our device and to join us in further developing DISC3D for archiving and 3D-modelling purposes. We will be happy to provide relevant information and share our experience.

## Supplementary materials

Supplementary material 1 Technical information Data type: text, figures and tables Explanation note: Detailed technical information and additional theoretical background. This is an open access article distributed under the terms of the Creative Commons Attribution License (CC BY 4.0), which permits unrestricted use, distribution, and reproduction in any medium, provided the original author and source are credited.Ströbel B, Schmelzle S, Blüthgen N, Heethoff M

Supplementary material 2 SV1: EDOF imaging Data type: video Explanation note: This video demonstrates the effect of the registered EDOF-calculation. This is an open access article distributed under the terms of the Creative Commons Attribution License (CC BY 4.0), which permits unrestricted use, distribution, and reproduction in any medium, provided the original author and source are credited.Ströbel B, Schmelzle S, Blüthgen N, Heethoff M

Supplementary material 3 SV2: Illustrative examples Data type: video Explanation note: Illustrative examples of insects and snail shell models generated with DISC3D. This is an open access article distributed under the terms of the Creative Commons Attribution License (CC BY 4.0), which permits unrestricted use, distribution, and reproduction in any medium, provided the original author and source are credited.Ströbel B, Schmelzle S, Blüthgen N, Heethoff M
