# Supplementary material for: An automated device for the digitization and 3D modelling of insects, combining extended-depth-of-field and all-side multi-view imaging
Source: Zookeys. 2018 May 17;(759):1–27. doi: 10.3897/zookeys.759.24584 (PMC5968080; doi:10.3897/zookeys.759.24584)
Supplement: Supplementary material 1 — Technical information [file zookeys-759-001-s001.docx]

**Supporting Information**

**An automated device for the digitization and 3D modeling of insects, combining extended-depth-of-field and all-side multi-view imaging**

Bernhard Ströbel^1^, Sebastian Schmelzle^2^, Nico Blüthgen^2^, Michael Heethoff^2,*^

^1^: Department of Mathematics and Natural Sciences, University of Applied Sciences Darmstadt, Schöfferstr. 5, 64295 Darmstadt, Germany

^2^: Ecological Networks, Technische Universität Darmstadt, Schnittspahnstr. 3, 64287 Darmstadt, Germany

^*^: Corresponding author: heethoff@bio.tu-darmstadt.de

**(S1) Choice and use of the optics**

The camera was chosen by the following considerations:

(i) The high number of shots needed for an insect scan (number of poses × number of stack images) precludes the use of a camera with a mechanical shutter. An "industrial camera" is preferable to a "consumer camera" because it is compact, fully configurable and controllable by software, and provides truly raw image data.

(ii) Since light abounds in the insect scanner, a CMOS sensor is preferable to CCD (higher frame rates, no smearing).

(iii) A color sensor is needed to image the insects in natural color.

(iv) Assuming that the StackShot macro rail is operated with its maximum speed of of 5 mm/s, a frame rate of at least 25 frames per second is desirable, allowing a minimum focal plane step size of 0.2 mm, appropriate for small specimens.

(v) As a very small angle of view is unfavorable for SfM, the sensor format should be at least 1/1.2" and preferably quadratic.

(vi) The number of pixels is of secondary importance for 3D modeling if shortcomings in focus stacking and 3D reconstruction exceed the pixel size. Note that a high number of pixels inflate measuring and processing time. For digitization without 3D modeling, however, a higher number of pixels would be preferable.

(vii) Since neither high-speed data throughput nor cable length is critical, the USB3 Vision camera bus was chosen.

At the time of the first setup of our insect scanner, the DFK 23UX174 camera of TheImagingSource Inc. proved to comply at the best with these criteria. It is equipped with a Sony Pregius IMX174 sensor with 1920 × 1200 pixels of *p* = 5.86 µm size (1/1.2" sensor format, long side 11.3 mm). Some of the models shown in this study were taken with that camera. Meanwhile, we use a Basler acA2040-90uc of Basler AG with a quadratic CMV4000 sensor featuring 2040 × 2046 pixels of *p* = 5.5 µm size and a sensor side length of 11.2 mm.

As for the lens, the criteria were:

(i) The lens should be corrected for a large sensor size and a close-up range up to *m* = 1. For magnifications *m* > 1, it should be mountable in reverse.

(ii) The lens must feature an adjustable diaphragm. A focusing ring or a zoom are not required; focusing is accomplished with extension tubes and the variation in the distance of the object.

(iii) A short focal length is recommended, as it results (together with a large sensor) in a larger angle of view, which is advantageous for the simultaneous camera calibration in the SfM method.

(iv) Compact dimensions of the lens body allow a good angular access to the underside of the pinned insects (a high negative elevation angle *α*, *cf*. Fig. S2).

Based on these considerations, we chose the Schneider Kreuznach Componon S12 V-mount macro lenses 2.8/28 for larger insects and 2.8/50 for smaller insects, together with Unifoc C-mount adapters to the camera and C-mount extension rings. With a diameter of the lens body of 47 mm this lens is much more compact than comparable high-class macro lenses for SLR cameras with typical diameters ≥ 73 mm.

In practice, there is a mechanical constraint to the working distance (from the front end of the lens to the insect) caused by the front light dome. With reference to Fig. S2, we estimate the maximum magnifications (and hence insect sizes) for the Componon lenses with our camera. We define the insect size *I* as diameter of its minimum bounding sphere (the smallest sphere that contains the insect as a whole). To image the insect from all sides, it is mounted on the gimbal with the center of that sphere in the gimbal center point CP, which is (ideally) the point of the mutually perpendicular intersection of the two gimbal axes and the camera axis. For maximum magnification, the minimum bounding sphere coincides with the "measuring range", the sphere around CP which is fully imaged on the quadratic sensor with side length *S* in any orientation of the gimbal. In this case, the magnification is:

*m = S/I* [1]

To provide sharp images of the entire insect for focus stacking, we must be able to move the focal plane (the plane of sharp imaging) through the whole measuring range from the near to the far end. In a simplified pinhole camera model, we assume that the lens is a "thin lens" with entrance and exit pupils coinciding in the center point of the lens body, the center of projection P. Then, the mechanical constraints of the length *L* of the lens body and the outer radius *R* of the front light dome result in a minimum object distance for sharp imaging:

*d > I/2 + R + L/2* [2]

The relationship between the object distance *d*, the fixed image distance or camera constant *c*, and the lens focal length *f*_L_ is given by the Gaussian lens formula

1/*d* + 1/*c* = 1/*f*_L_ [3]

We solve Eq. [3] for *d*, eliminating *c* = *m* ∙ *d*:

*d* = *f*_L_ ∙ (1 + 1/*m*) [4]

Inserting *I* from Eq. [1] into Eq. [2] and equalizing Eqs. [2] and [4], we obtain:

*f*_L_ ∙ (1 + 1/*m*) > *S*/2*m* + *R* + *L*/2 [5]

Solving for *m*, we obtain the maximum achievable magnification given the mechanical constraint:

*m*_max_ *=* $\frac{f_{L}-S/2}{R+L/2-f_{L}}$ [6]

With *R* = 62 mm (radius of our front light dome) and *S* = 11.2 mm (sensor side length), we calculate *m*_max_ = 0.44 for the Componon 2.8/28 lens (with length *L* = 33 mm and diameter *D* = 47 mm) and *m*_max_ = 1.41 for the Componon 2.8/50 lens (*L* = 39 mm, *D* = 47 mm) respectively. The maximum negative elevation angle is *α* = -arctan(2*R*/*D*) ≈ -70° in both cases. In practice, we exclude negative elevation angles < -70° as well as positive angles >70° from our pose programs, the former for the said machanical constraint, the latter for to prevent part of the gimbal from appearing in the background of the specimens. This restriction reduces the number of usable poses in our standard pose program with 10° angular distance from 412 to 398, which still covers 96.6 % of all spatial directions.

**(S2) Depth of field (DOF) calculation and focus stacking for insect scanning**

Let *f*_L_ be the lens focal length, *N*_L_ the lens f - number (*f*/#), *m* the magnification (here defined as an unsigned number), and *B*_defocus_ the accepted defocus blur in the image space, also referred to as the diameter of the "circle of confusion". Assuming a magnification in the order of 1 (let's say between 0.1 and 10), and further assuming that *B*_defocus_<< *m*·*D*_EP_, where *D*_EP_ = *f*_L_/*N* _L_ is the diameter of the lens entrance pupil, while neglecting effects of lens asymmetry, we obtain based on geometric optics (Conrad 2006; Greivenkamp 2004; Larmore 1965; Ray 2002) for the depth of field, i.e. the object depth range where the defocus blur is *B*_defocus_ or less:

*DOF = 2N*_L_ *∙ B*_defocus_ *∙* $\frac{m+1}{m^{2}}$ [7]

To avoid the resolution of the digital image being affected, the defocus blur on the sensor should not exceed twice the sensor pixel size (side length) *P*_S_:

*B*_defocus_ ≤ 2*P*_S_, [8]

in accordance with the Nyquist criterion. If enough light is available, Eq. [7] suggests that the DOF can be extended by stopping down the lens, if necessary even by integrating an additional pinhole. But stopping down introduces diffraction blur which can be quantified by the diameter of the Airy disk on the sensor (Greivenkamp 2004):

*B*_diffract_ = 0.5 · *D*_Airy_ = 1.22*N* _L_ · *λ* · (*m*+1), [9]

where *λ* = 0.56 µm is the central wavelength of the visible light. The factor 0.5 considers the soft profile of the Airy disk, compared to the circle of confusion for defocus. As before, we assume that the diffraction blur is not noticeable as long as *B*_diffract_ does not exceed twice the pixel size, which is the Rayleigh criterion for the resolution of two point-like objects:

*B*_diffract_ ≤ 2*P*_S_  [10]

Eq. [10] implies an optimum *f* - number for maximum DOF with no noticeable impairment of the image resolution by additional diffraction:

*N*_L opt_ *=* $\frac{P_{S}}{0.61\lambda\cdot\left( m+1 \right)}$ [11]

Inserting Eqs. [8] and [11] into Eq. [7] we obtain the depth of field for optimum resolution of the unprocessed digital image, in the absence of further image degradation due to aberrations, motion, sensor noise, etc.:

*DOF =*$\frac{6.56 P_{S}^{2}}{\lambda\cdot m^{2}}$ [12]

Introducing the object (or insect) pixel size *P*_I_ = *P*_S_ / *m*, a quantity of prime interest to the user which will henceforth be referred to as “digital resolution”, we can reformulate Eq. [12]:

*DOF* =$\frac{6.56 P_{I}^{2}}{\lambda}$ [13]

We now assume that an insect of size *I* is imaged “full frame” with the magnification *m* on a quadratic sensor of sidelength *mI* and area (*mI*)². We replace in Eq. [12] *P*_S_² = *I*² / *N*_pix_ where *N*_pix_ is the number of pixels on the sensor, and *DOF* = *I* / *N*_FS_ where *N*_FS_ is the number of frames (images) needed for focus stacking of the insect. If we solve for *N*_FS_ we get:

*N*_FS_ *=*$\frac{N_{\mathrm{pix}} \cdot\lambda}{6.56 I}$ [14]

The focus stacking parameters applied for the three optical configurations used in this study are compiled in Table S1.

Eqs. [13] and [14] give two useful rules of thumb:

1. An insect can be imaged with the digital resolution *P*_I_, given in micrometers, without noticeable blurring by defocus or diffraction, within the following depth-of-field, also in micrometers:

(*DOF*/µm) ≈ 12 × (*P*_I_/µm)² [15]

Example: A resolution (= insect pixel size) of 10 µm allows sharp imaging within a depth-of-field of 1200 µm or 1.2 mm.

1. Imaging of an insect of the size *I*, given in millimeters, full frame on a quadratic sensor with *N*_pix_ pixels, given in megapixels, without noticeable blurring by defocus or diffraction, requires focus stacking with at least the following number of frames:

*N*_FS_ ≈ 85 ×$\frac{({N_{\mathrm{pix}}}/\mathrm{MP})}{(I/\mathrm{mm})}$ [16]

Example: To image a 20 mm long insect full frame with extended depth-of-field on a 4 MP sensor, focus stacking with at least 17 frames is recommended.

**(S3) Macro rail**

A StackShot automated macro rail system of Cognisys Inc. is used to take images for focus stacking. In "continuous mode", the device moves the camera continuously with *v* = 5 mm/s (the maximum velocity) in axial direction towards the specimen over a predetermined travel distance that is set to a value 3 mm larger than the insect size *I*. The forward motion is followed by an automatic return to the starting point with the same velocity. Together with the use of a stepper motor, this results in the high reproducibility required for the calibration process described in the following section. During the forward motion, the camera takes frames under front light with an appropriately chosen video frame rate *n* resulting in a focus stacking step size of

Δ*z* = *v* / *n*. [17]

The axial motion of the camera during exposure is neglectable (50 µm for a typical exposure time of 10 ms) and has no influence on image quality. During the backward motion, frames are taken under back light at the same rate. As the macro rail accelerates during the first and decelerates during the last millimeter, the first and last millimeters are not useable for the focus stack evaluation. The macro rail parameters applied in this study can be found in Table S1.

It is important that the camera axis is aligned with the direction of the macro rail motion, as a deviation of the two directions causes a shift between the near and the far parts of the imaged specimen which could impair the quality of the 3D model. For this reason, an adjusting device has been included in the mounting of the camera on the macro rail (see Fig. 3).

**(S4) Calibration and implementation of the focus stacking process**

For calibration of the focus stacking process, a flat dot matrix target, featuring a regular grid of 6 × 8 circular black marks on a white ground, is placed in the plane through the center point CP and perpendicular to the camera axis. At first, the camera is adjusted for a sharp image of the dot matrix; this position of the camera is named "reference position" (Fig. S3a). The related "reference magnification" *m*(0) can be calculated from the known size *T* of some feature on the dotmatrix (in mm), and the size *T'*(0) of that fearure on the image (in pixels, with known pixel size in mm). It is equal to the quotient of the camera constant *c* and the object distance for sharp imaging *d*:

*m*(0) = *T '*(0) / *T* = *c* / *d* [18]

Then, by aid of the macro rail, the camera is moved forth and back around the reference position, with a total distance exceeding the the maximum size *I* of the insects to be scanned with the magnification *m*(0):

*I* = *S* / *m*(0) [19]

where *S* is the size (side length) of the sensor. During the camera motion, a video sequence is recorded in the same way as will be later done in the insect scan. To minimize the defocus blur during calibration, the images of the target are taken with the smallest possible aperture. Two image stacks ("focus stacks") are extracted from the video sequence, one with the camera in uniform forward motion, and one in uniform backward motion. For all images of the stacks, the centers of the (albeit blurry) marks are evaluated to determine (i) the components *t*_x_ and *t*_y_ of the image shift (in pixels) with respect to the image in the reference position, and (ii) the magnifications of the images of the dot matrix. This data will later be used to "register" the insect focus stacks, i.e. to shift and scale the images to make them superimposable, which is the first step for the calculation of the image with extended depth-of-field (EDOF image).

As for the shift components, we usually find a linear dependence on the camera position, originating from the remaining non-parallelism of the camera axis and the direction of the macro rail motion, superimposed with a portion periodic with the pitch of the threaded control rod of the macro rail mechanics.

From the magnifications of the target images, both, the scaling factors to be applied for the registration of the insect images, and important parameters of the camera configuration can be found: Let *z* be the camera position for some stack image, defined as the signed deviation from the reference position, with a positive sign for positions farther away from the object. In position *z*, the magnification of the blurry target image on the sensor is:

*m*(*z*) = *T '*(*z*) / *T* = *c* / (*d* + *z*) [20]

according to ray optics (*cf*. Fig. S3b). In particular, the magnifications *m*_near_ = *m*(*z*_near_) and *m*_far_ = *m*(*z*_far_) at the nearest and the farthest point of the macro rail travel are evaluated. Then, by elimination of *d*, the camera constant *c* can be calculated from Eq. S20 by use of the well-known one-way travel distance *Z*_travel_ = *z*_far_ - *z*_near_ of the macro rail:

*c* = *Z*_travel_ · *m*_near_ · *m*_far_ / (*m*_near_ - *m*_far_) [21]

In contrast to the flat dotmatrix target, an insect is a 3D object. When a camera moves towards a 3D object, the magnifications of the different portions of the image grow by different factors, i.e. the image is not just "zoomed" but its perspective changes. In the strict sense, as each image in the stack has a different perspective, it is impossible to scale them all for a fully correct registration. However, since only the sharp portions of the images will become part of the EDOF image, a fully correct registration is unnecessary. Fig. S3c shows that, with the camera in position *z*, an insect structure *J* at position *z* (with respect to CP) is in focus, giving a sharp image of the size

*J'*(*z*) = *J* · *c* / *d*. [22]

This sharp image of the structure *J* will be used in the EDOF image. For the subsequent 3D reconstruction, the EDOF image must have a unique perspective, in other words, it must comply with the pinhole camera model. The natural choice for the common perspective is that of the camera in the reference position (Fig. S3d). In this position, the structure *J* is imaged blurry with size *J"* in the sensor plane:

*J"*(*z*) = *J* · *c* / (*d* - *z*) [23]

Therefore, the stack image taken at the camera position *z* must be rescaled to bring the sharp image of structure *J* from the size *J'* to the size *J''*. The required scaling factor *sc* can be calculated from Eqs. [18], [20], [22], and [23]:

*sc*(*z*) = *J''*(*z*) / *J'*(*z*) = *d* / (*d* - *z*) = $\frac{1}{2 - m\left( 0 \right)/m(z)}$ [24]

The EDOF image will have the reference magnification $m$(0), and its projection center P will be in the reference distance

$d= \frac{c}{m\left( 0 \right)}=Z_{\mathrm{travel}}\cdot\frac{m_{\mathrm{near}} \cdot m_{\mathrm{far}}}{m\left( 0 \right) \cdot\left( m_{\mathrm{near}}-m_{\mathrm{far}} \right)}$ [25]

from the center point CP (*cf*. Eqs. [18] and [21]). This quantity will later be used to calibrate the scaling of the 3D model in the SfM calculation (see S7).

During an insect scan, the time required to record an image stack (with forward and backward motion of the macro rail carriage) is used to evaluate the previous stack. With regard to computational efficiency and minimization of interpolation errors, the registration (i.e. translation and scaling with linear interpolation) of the recorded images is performed for both, columns and rows, by one single sparse matrix multiplication each. The sparse matrices needed for this operation are calculated subsequent to the stack calibration from the obtained registration parameters *t*_x_, *t*_y_, and *sc*, and are stored in a cell array for all stack images to be evaluated.

For the described calibration of the focus stacking process, it is vital that the images are repeatably taken at the same positions of the camera. The first "exposure active" (flash sync) output signal of the camera triggers the UP button of the StackShot controller via a relais, and thereby synchronizes the macro rail motion with the video stream (*cf*. Fig. 1A).

Once the stack images are registered, blending, i.e. an additive superposition weighted by the local sharpness of the images, is performed to generate the final EDOF image. The stack images are converted to grayscale and subject to a filtering process which asseses the local sharpness. Two different metrics of local sharpness were used: (i) difference of Gaussian (DoG) high pass filtering, followed by Gaussian low pass filtering of the absolute value, and (ii) calculation of local variance, also followed by Gaussian low pass filtering. The filter parameters can be optimized interactively with respect to the local and overall sharpness of the EDOF image, calculation time, and onset of artifacts. For the weighting factor, the local sharpness measure is raised to a power *p*, which allows for different degrees of averaging between neighboring stack images. Low values of *p* (< 8) trend to have a more averaging character of the blending with slightly blurred EDOF images, while high values (>12) give a more stitching character with the possibility of local artifacts.

As a by-product of stack blending, we obtain a "depth image" (or range image) representing the position of maximum sharpness for every pixel within the stack. Although this image contains 3D information, it is not used for the subsequent generation of the 3D model, since both its lateral and its depth resolutions are relatively coarse. Nevertheless, a pseudocolor encoded depth image is helpful as a guide when interactively optimizing the blending parameters. The whole process of stack evaluation, including registration and blending, is shown schematically in Fig. 4.

The EDOF image can be further enhanced by gamma correction, contrast stretching, and modification of the color saturation. These operations can considerably increase the number of detectable feature points, but the user should primarily have the features in the "difficult" parts of the insect bodies (dark, even, poorly textured) in mind, rather than the total number of features. If the result of the scan is a colored and textured 3D model, the natural appearance of the insects should not be modified too much.

**(S5) Background Masking**

Whereas the front light EDOF images are the actual base of the 3D modeling with SfM, the back light EDOF images merely serve for masking the former to exclude any background features from feature matching. The back light EDOF images are first converted to grayscale and divided by an "empty" back light image (image without an insect) in order to compensate for the not fully uniform background illumination. Also, spurious front light illumination, originating from backscatter by the front light dome has to be determined and removed. The negative of the resulting grayscale image is essentially an opacity image: within the bulk of the insect body, the opacity has its maximum value (shown white), whereas the transparent parts of the body like wings and hairs show a lesser degree of opacity. A binary mask (1 in the object, 0 in the background) is generated by interactive definition of an opacity threshold. In this way, the user can keep the wings intact, and at the same time exclude halo artifacts from becoming parts of the object. A masked front light EDOF image (final image) from every pose reached during the scan is transferred to the SfM software in the PNG format. The mask information is coded in the 8-bit alpha channel of this image: for object pixels the alpha value is set to 255, for background pixels to 0. The whole masking process is shown schematically in Fig. 5.

**(S6) Optical configurations, scan parameters and scan times**

Most insect scans presented in this study were taken in one of the following optical configurations:

- larger insects (16 mm ≤ *I* ≤ 27 mm) with the Componon 2.8/28 lens in normal orientation and *m* = 0.41
- medium sized insects (9 mm ≤ *I* ≤ 16 mm) with the Componon 2.8/50 lens in normal orientation and *m* = 0.72
- small insects (*I* ≤ 9 mm) with the Componon 2.8/50 lens in reversed orientation and *m* = 1.26

The optical, mechanical, and scan parameters of these configurations are compiled in Table S1.

All configurations use the Basler acA2040-90uc camera equipped with a quadratic CMV4000 sensor featuring 2040 × 2046 pixels with *P*_S_ = 5.5 µm pixel side length and *S* = 11.2 mm sensor side length. Further values entering the calculations are the central wavelength of visible light *λ* = 0.56 µm, the macro rail velocity *v* = 5 mm/s, and our standard number of poses per scan *N*_P_ = 398. The scan times given in the table were found for an Intel Pentium i7-4770 (3.4GHz) system with 32GB RAM and Nvidia Geforce GTX 650 Ti graphics. These times could be reduced with more powerful hardware, but never lower than the frame recording time. The use of parallel computing options in MATLAB turned out to be less effective, due to the high amount of data transfer between the processor cores.

Whereas most of the parameters shown in Table S1 originate in the optical and mechanical constraints of the setup, two can be freely modified with great influence on both, scan time, and quality of the resulting EDOF images and 3D models: (i) the video frame rate *n* which determines the focus stacking step size Δ*z* via Eq. [17], and which is only limited by the maximum frame rate of the camera of 90 s^-1^, and (ii) the lens *f* - number *N*_L_ which can be chosen between 2.8 (open diaphragm, marked with 1) and 16 (closed diaphragm, marked with 6). It seems obvious to choose *N*_L_ from Eq. [11] and to adapt Δ*z* = *v* / *n* to the DOF calculated from Eq. [12]. But the derivation of these formulae is to some degree discretionary (Eqs. [8] - [10]), so that a detailed empirical study considering the trade-off between scan time and quality of the results for the different types of insect bodies (compact or delicate, little or much texture, light-colored or dark-colored) has still to be accomplished. The values shown in Table S1 are those used for most of the examples shown in this study.

Another parameter with huge influence on both scan time and quality is the number of poses *N*_P_. Doubling the angular distance from 10° to 20° would reduce the number of poses from ca. 400 to ca. 100, reducing the full-frame scan times to about one hour. A future empirical study considering the different shapes of the insect bodies and the desired degree of 3D detail is needed as well. All scan times shown in Table S1 refer to *N*_P_ = 398.

For insects not occupying the full frame size of the respective optical configuration, our software gives options for 3/4 size (with 1536 × 1536 pixels) and half size (with 1024 × 1024 pixels) evaluation. In these cases, not only the evaluated image region, but also the number of evaluated stack frames is reduced. The macro rail travel distance and hence the recording time are kept unchanged because the focus stacking calibration is associated with a particular macro rail motion.

**(S7) Camera calibration and absolute scaling of the 3D model**

The SfM calculation with Agisoft PhotoScan Pro is based on the "internal orientation" of the camera, in particular the camera constant given in sensor pixels *c* / *P*_S_. We obtain this parameter, which is often named "focal length" as it can be regarded as the focal length in the pinhole camera model, from the focus stacking calibration (see S4). The other parameters of the internal orientation (distortion etc.) are not determined and seem not to be of any major relevance for the 3D modeling of insects with the lenses used.

For the absolute scaling of the 3D data (point clouds and models), the positions of the cameras in the object coordinate system are calculated for all poses, using the reference distance *d* from Eq. [25], and the elevation and azimuth angles of the gimbal as defined by the pose program. These positions are transferred to PhotoScan Pro as reference positions for the absolute scaling of the 3D data. The positions calculated by PhotoScan Pro from the simultaneous camera calibration ("external orientation") show systematic deviations in the order of magnitude of 0.5 mm from the reference positions, which are due to the imperfectness of the gimbal. By repetitive elimination of weak points from the point cloud and optimization of the camera parameters, with all internal parameters besides the focal length constrained to zero, the "total error" of the points can be reduced. It was found that the inclusion of some or all the other internal parameters could not further reduce the error.

The diameter of the sphere can be evaluated from scans with the textured sphere target (see Fig. 6), giving an independent verification of the absolute scaling. It was repetitively found that the value of the diameter, as determined from the point cloud with a scaling based on the reference distances *d* and hence on the macro rail travel distance *Z*_travel_ (see Eq. [25]), deviates from the diameter measured with the caliper by less than 1%. Therefore, we assume for the distances measured on our 3D models an uncertainity of ±1 %. When the camera parameters calibrated with the textured sphere target are used to calculate the point cloud of an insect ("pose calibration"), the "fix calibration" option of PhotoScan Pro should be chosen, because any change of the focal length parameter would also change the model scaling.

**Table S1: Data for the optical configurations used in this study**

| camera configuration, depending on insect size: | large | medium | small |
| --- | --- | --- | --- |
| lens | 2.8/28 | 2.8/50 | 2.8/50 R |
| lens body length *L* | 33 mm | 39 mm | 39 mm |
| extension rings (incl. C-mount adapter) | 19 mm | 59 mm | 91.5 mm |
| camera constant *c* | 41 mm | 89 mm | 109 mm |
| pixel-based pinhole camera model focal length *f* = *c*/*P*_S_ | 7450 | 16200 | 19800 |
| object distance for sharp imaging *d* ^(1)^ | 100 mm | 124 mm | 87 mm |
| lens focal length *f*_L_= (1/*c* + 1/*d*)^-1^ | 29 mm | 52 mm | 48 mm |
| magnification for sharp imaging *m* = *c*/*d* ^(2)^ | 0.41 | 0.72 | 1.26 |
| field of view (FOV) side length *S*/*m* | 27.4 mm | 15.6 mm | 8.9 mm |
| macro rail one-way travel distance *Z*_travel_ | 30 mm | 19 mm | 12 mm |
| depth of the evaluated focus stack *Z*_eval_ ^(3)^ | 28 mm | 17 mm | 10 mm |
| maximum insect size *I* | ca. 27 mm | ca. 16 mm | ca. 9 mm |
| free space in the nearest camera position ^(4)^ | 2 mm | 36 mm | 2 mm |
| frame recording time per pose *t*_rec_ ^(5)^ | 13 s | 8.6 s | 5.8 s |
| frame recording time for a complete scan *N*_P_ ‧ *t*_rec_ | 1.44 h | 0.95 h | 0.64 h |
| video frame rate *n* ^(6)^ | 3 s^-1^ | 8 s^-1^ | 20 s^-1^ |
| number of frames recorded per pose *N*_F rec_ = *n* ‧ *t*_rec_ | 39 | 69 | 116 |
| raw data volume per scan *N*_P_ ‧*N*_F rec_ ‧2040 ‧2048 ‧3 byte | 0.19 TB | 0.43 TB | 0.57 TB |
| number of frames evaluated per pose *N*_F eval_ ^(7)^ | 2 × 17 | 2 × 27 | 2 × 40 |
| focus stacking step size Δ*z* = *v* / *n* | 1.67 mm | 0.625 mm | 0.25 mm |
| calculated optimum *f* - number *N*_L opt_ ^(8)^ | 11.4 | 9.4 | 7.1 |
| calculated depth-of-field *DOF* ^(9)^ | 2.4 mm | 0.68 mm | 0.22 mm |
| found total scan time for full size evaluation | 3.4 h | 3.8 h | 4.9 h |
| found total scan time for 3/4 size evaluation | 2.6 h | 2.6 h | 3.0 h |
| found total scan time for 1/2 size evaluation | 2.1 h | 1.9 h | 1.7 h |

Annotations:

^(1)^ reference distance, distance of the EDOF image projection center P from the center point CP

^(2)^ reference magnification *m*(0), magnification of the central plane of the EDOF image

^(3)^ both, first and last millimeter of the travel distance *Z*_travel_ are used for acceleration and deceleration and are thus not evaluated. The evaluated depth *Z*_stack_ slightly exceeds the FOV side length, to accommodate for protruding parts of the insect bodies.

^(4)^ space between the lens body and the front-light dome

^(5)^ frames are recorded during the whole macro rail round-trip: 2 ‧ *Z*_travel_ / *v* for the motion with velocity *v* plus plus 1 s for acceleration, deceleration, and interim stoppage

^(6)^ chosen to match the focus stepping step size to the DOF, while for the "small" configuration a lower value was chosen to confine the number of frames and hence the scan time

^(7)^ only the frames recorded during the uniform forward and backward motion of the macro rail are evaluated: *N*_F eval_ = 2 ‧ *n* ‧ *Z*_eval_ / *v*; the number is reduced for 3/4 size and half size evaluation

^(8)^ as calculated from Eq. [11]; in practice, a somewhat lower *f* - number gave slightly sharper results

^(9)^ as calculated from eq. [12]; *cf*. with the focal plane step size Δ*z*


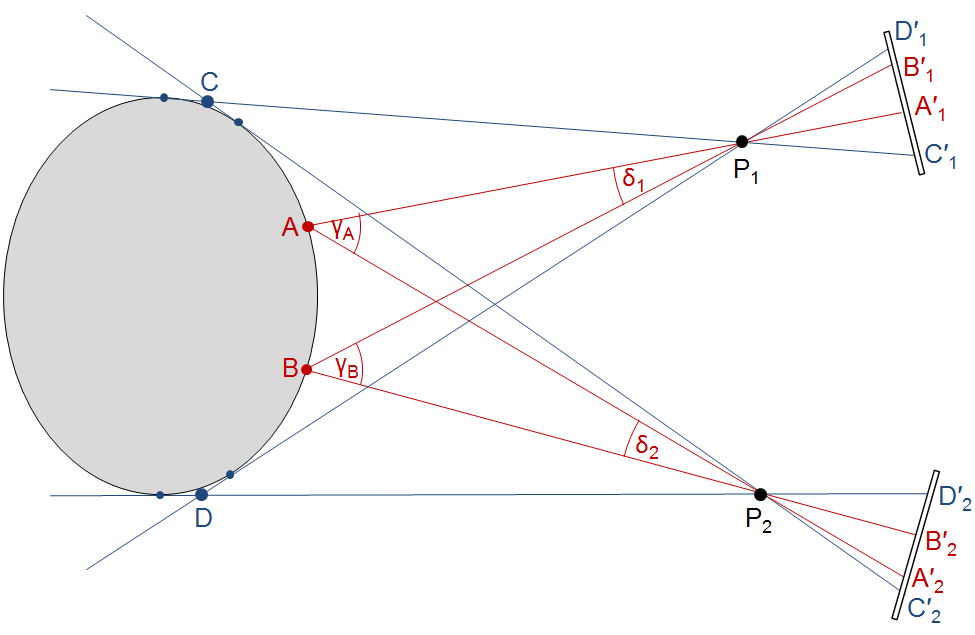


**Figure S1** Comparison of the triangulation geometry for the "Structure from Motion" (red) and "Shape from Silhouette" (blue) techniques, assuming two cameras (P_1_ and P_2_).

SfM uses viewing rays starting from feature points (A, B) on the object surface. By matching their images on the sensors of both cameras (A'_1_ and A'_2_, B'_1_ and B'_2_, and many others), it is possible to determine the spatial locations of the feature points and of the camera projection centers and orientations simultaneously. In contrast, SfS is based on the intersection of the visual cones of the object with respect to the cameras, the so-called "visual hull". The viewing rays are here tangential to the object, and their intersections (C, D), defining the visual hull, are not points on the object surface. Therefore, matches cannot be found, and it is not possible to perform a simultaneous calibration of the cameras.


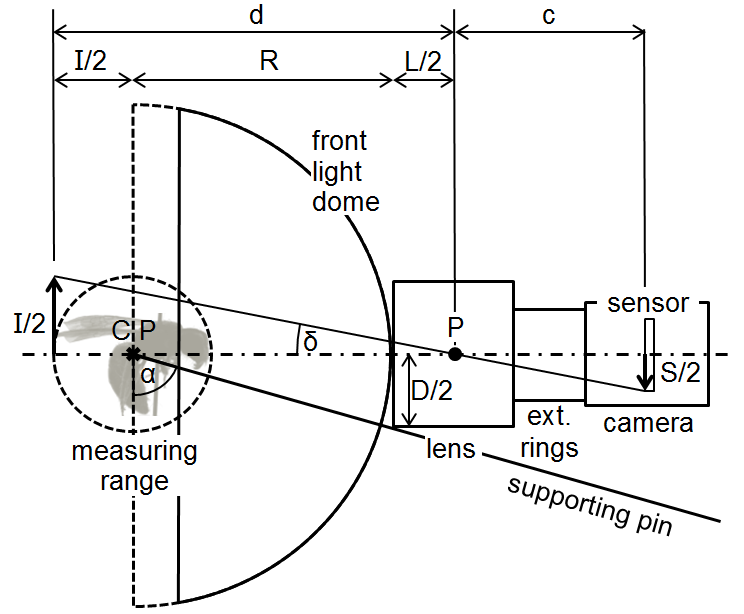


**Figure S2** **Constraint on the camera position by the front light dome**

Camera and lens are shown schematically in the nearest possible position of the macro rail, where the lens body just touches the front light dome. Then, in the limiting case, the far end of the measuring range has a sharp image on the sensor. The center of projection P in the pinhole camera model is assumed to be in the center of the lens body. From these simplified assumptions, the maximum achievable magnification with a given camera and lens combination can be calculated, as being developed in the text.

***
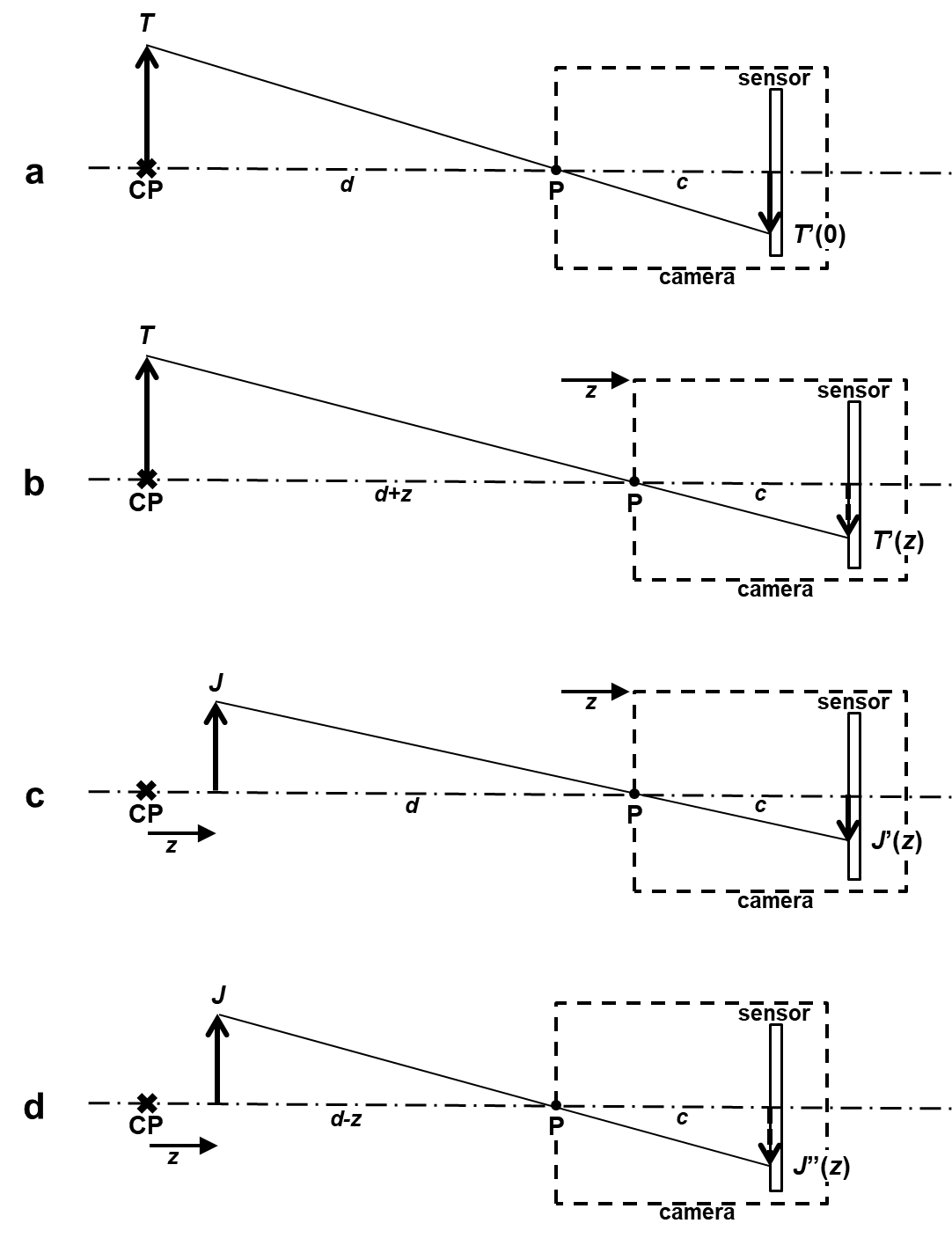
***

**Figure S3** **Calibration of focus stacking**

(a) A flat target *T* is placed in the center point CP of the gimbal, and the camera is positioned for a sharp image *T'* (0) on the sensor. This position of the camera is the "reference position".

(b) The camera is moved to different positions *z*, and the positions and sizes of the blurry images *T'*(*z*) on the sensor are recorded.

(c) During an insect scan with the camera in position *z*, the insect structure *J* with the offset *z* against the CP, gives a sharp image of size *J'*(*z*).

(d) For the composite EDOF image, *J'* has to be rescaled to the size *J''* of the blurry image of *J* in the reference position. The scaling factor can be calculated from *T'*(*z*) and *T'*(0).

**References**

Conrad J (2006) Depth of field in depth. wwwlargeformatphotographyinfo/articles/DoFinDepthpdf:

Greivenkamp JE (2004) Field Guide to Geometric Optics. SPIE Press, Bellingham, USA, 128 pp.

Larmore L (1965) Introduction to Photographic Principles. Dover Publications, New York, 229 pp.

Ray SF (2002) Applied Photographic Optics. Focal Press, Oxford, 680 pp.
